# The Role of Epithelial–Mesenchymal Transition in Malignant Pleural Mesothelioma: From Pathogenesis to Diagnosis and Treatment

**DOI:** 10.3390/cells14080585

**Published:** 2025-04-12

**Authors:** Dimitrios E. Magouliotis, Fabrizio Minervini, Ugo Cioffi, Matilde De Simone, Davide Patrini, Marco Scarci

**Affiliations:** 1 Department of Cardiac Surgery Research, Lankenau Institute for Medical Research, Main Line Health, Wynnewood, PA 19096, USA; dimitrios.magouliotis.18@alumni.ucl.ac.uk; 2 Luzern Kanton Hospital, 6000 Luzern, Switzerland; fabriziominervini@hotmail.com; 3 Department of Surgery, University of Milan, 20122 Milan, Italy; ugo.cioffi@guest.unimi.it (U.C.); matilde.desimone@guest.unimi.it (M.D.S.); 4 University College of London Hospital, London NW1 2BU, UK; davide.patrini@nhs.net; 5 Department of Cardiothoracic Surgery, Hammersmith Hospital, Imperial College Healthcare, National Health Service (NHS) Trust, London W2 1NY, UK

**Keywords:** epithelial–mesenchymal transition, EMT, malignant pleural mesothelioma, MPM, immunotherapy, biomarkers, clinical trials

## Abstract

Malignant pleural mesothelioma (MPM) is an aggressive cancer of the pleural lining, primarily associated with asbestos exposure. Despite advancements in multimodal treatment, patient survival remains poor. Epithelial–mesenchymal transition (EMT) has emerged as a crucial process driving MPM pathogenesis, metastasis, and resistance to therapy. This review explores the molecular mechanisms underlying EMT in MPM, including key signaling pathways such as TGF-β, Wnt/β-catenin, and PI3K/Akt. We also discuss the diagnostic and prognostic significance of EMT-related biomarkers and emerging targeted therapies aimed at reversing EMT or exploiting EMT-induced vulnerabilities. Additionally, recent clinical trials, including the MARS 2 trial, are reviewed to provide insight into the evolving treatment landscape.

## 1. Introduction

Malignant pleural mesothelioma (MPM) is a rare but aggressive neoplasm with a poor prognosis [1]. It primarily arises from the mesothelial cells lining the pleura and is strongly linked to asbestos exposure [2]. The latency period between exposure and disease manifestation can be several decades, making early detection challenging [2]. Due to its aggressive nature and late-stage diagnosis, the treatment of MPM remains a significant challenge, with most patients surviving only 12 to 18 months after diagnosis despite aggressive multimodal therapy [1]. The disease is histologically classified into three main subtypes: epithelioid, sarcomatoid, and biphasic, with the epithelioid type being associated with a better prognosis.

Current treatment strategies include chemotherapy, immunotherapy, and surgery, yet their effectiveness varies significantly among patients. Chemotherapy regimens based on pemetrexed and cisplatin have been the mainstay of treatment, but they offer only modest survival benefits. Immunotherapy with checkpoint inhibitors such as nivolumab and ipilimumab has demonstrated efficacy, particularly in non-epithelioid MPM, leading to their approval as frontline treatment options. However, despite these advances, MPM remains a highly resistant and recurrent malignancy, emphasizing the need for novel therapeutic targets.

EMT has emerged as a critical biological process in the progression of MPM, enabling tumor cells to acquire invasive and metastatic properties [3]. EMT is characterized by the loss of epithelial features, such as cell–cell adhesion, and the gain of mesenchymal traits that enhance motility and resistance to apoptosis. This phenomenon plays a significant role in tumor progression, treatment resistance, and immune evasion. Understanding the molecular mechanisms underlying EMT in MPM may provide new avenues for therapeutic intervention, particularly in addressing the treatment resistance associated with this disease [2].

This review aims to comprehensively explore the role of EMT in MPM pathogenesis, its impact on tumor progression and treatment resistance, and its significance as a diagnostic and prognostic biomarker. Beyond mesothelioma, EMT plays a critical role in the pathogenesis of many epithelial cancers, including breast, colorectal, and lung carcinomas [4]. EMT enables cancer cells to detach from the primary tumor, invade surrounding tissues, and enter circulation, facilitating metastasis [4]. This transition is driven by complex signaling cascades that activate mesenchymal markers while repressing epithelial characteristics, ultimately conferring increased motility, drug resistance, and stem-like properties to tumor cells [5]. Additionally, emerging therapeutic strategies targeting EMT-related pathways are discussed, along with insights from recent clinical trials, including the MARS 2 study, which has sparked debate over the role of surgery in MPM management.

## 2. EMT in MPM Pathogenesis

**Figure 1:**
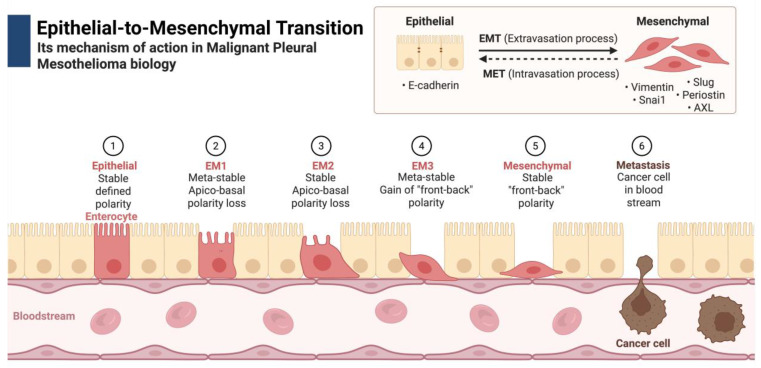
Schematic representation of the epithelial-to-mesenchymal transition (EMT) mechanism in malignant pleural mesothelioma. The illustration highlights key molecular events, including the loss of epithelial markers, acquisition of mesenchymal traits, and activation of signaling pathways that drive tumor progression, invasion, and resistance to therapy. Created in BioRender. Magouliotis, D. (2025) https://BioRender.com/p74t526 (accessed on 10 April 2025).

Epithelial–mesenchymal transition (EMT) is a process by which epithelial cells lose their polarity and adhesion properties, acquiring a mesenchymal phenotype characterized by increased motility and invasiveness (Figure 1) [2]. This transformation is particularly relevant in the pathogenesis of MPM, where EMT contributes to tumor progression, metastasis, and resistance to conventional therapies. The ability of MPM cells to transition between epithelial and mesenchymal states enables them to evade the immune system and develop resistance to treatment modalities, further complicating disease management.

The molecular regulation of EMT in MPM involves several key transcription factors, including SNAIL, TWIST, and ZEB [6]. These transcription factors repress epithelial markers such as E-cadherin while upregulating mesenchymal markers such as N-cadherin and vimentin. The loss of E-cadherin disrupts cell–cell adhesion, facilitating tumor cell dissemination [7]. Additionally, SNAIL and TWIST have been shown to enhance the expression of matrix metalloproteinases (MMPs), which degrade extracellular matrix components and promote invasion and metastasis [8].

Multiple signaling pathways regulate EMT in MPM (Table 1), with the TGF-β pathway being one of the most prominent [9,10,11]. TGF-β signaling induces EMT through both SMAD-dependent and SMAD-independent mechanisms, leading to the activation of mesenchymal genes and the suppression of epithelial markers. The Wnt/β-catenin pathway is another key player in EMT regulation, stabilizing β-catenin and promoting the transcription of EMT-related genes. The PI3K/Akt pathway further contributes to EMT by inhibiting GSK-3β, a key regulator of β-catenin degradation, thereby sustaining mesenchymal transformation [12,13].

The tumor microenvironment plays a significant role in EMT induction in MPM. Hypoxia, chronic inflammation, and interactions with tumor-associated fibroblasts and macrophages create a microenvironment conducive to EMT [5,14]. Hypoxia-inducible factors (HIFs) can upregulate EMT transcription factors, further promoting the mesenchymal phenotype. Tumor-associated fibroblasts secrete cytokines such as TGF-β and IL-6, reinforcing EMT signaling and supporting tumor progression [5,14]. These microenvironmental factors, in combination with intrinsic tumor signaling pathways, drive EMT in MPM and contribute to its aggressive behavior.

Notably, NF2 (neurofibromin 2), one of the most frequently mutated genes in mesothelioma, has been implicated in EMT regulation. NF2 loss promotes the activation of the Hippo–YAP/TAZ pathway, which in turn upregulates EMT transcription factors such as SNAIL and ZEB1 [15,16]. Studies have shown that NF2 inactivation enhances mesenchymal traits, suggesting its potential as a biomarker for EMT-driven disease subtypes and a possible therapeutic target [15,16].

## 3. Impact of EMT on MPM Progression and Treatment Resistance

**Figure 2:**
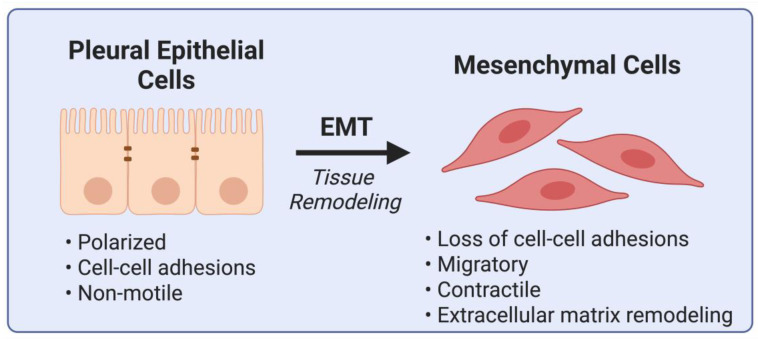
Schematic representation of tissue remodeling in malignant pleural mesothelioma. The figure illustrates the dynamic interactions between tumor cells, the extracellular matrix, and stromal components, emphasizing fibrosis, angiogenesis, and immune modulation as critical contributors to mesothelioma pathophysiology. Created in BioRender. Magouliotis, D. (2025) https://BioRender.com/w35w967 (accessed on 10 April 2025).

EMT plays a crucial role in the progression of MPM by facilitating tumor invasion and metastasis (Figure 2). EMT is not merely a phenotypic change in MPM; it has profound functional consequences that exacerbate disease progression. Through EMT, MPM cells acquire enhanced invasive and migratory capabilities, contributing to both local spread and distant metastasis. Table 2 highlights the key EMT biomarkers in solid tumors, including MPM. EMT-upregulated enzymes like MMP-2 and MMP-9 degrade extracellular matrix barriers, facilitating tumor cells’ penetration into surrounding tissues and the bloodstream [17]. Tumor cells that undergo EMT acquire a more motile phenotype, allowing them to disseminate within the pleural cavity and, in rare cases, metastasize to distant organs. EMT-driven MPM cells exhibit increased resistance to apoptosis, enabling them to survive under adverse conditions and resist immune surveillance. The enhanced invasive properties of mesenchymal-like tumor cells contribute to the aggressive nature of MPM and its poor prognosis [1].

One of the major consequences of EMT in MPM is the development of resistance to chemotherapy [18]. Standard chemotherapy regimens, such as pemetrexed and cisplatin, are less effective against mesenchymal-like tumor cells due to the upregulation of drug efflux pumps and activation of survival pathways. EMT-induced activation of the PI3K/Akt and NF-κB pathways further promotes chemoresistance by enhancing cell survival and inhibiting apoptotic signaling [19]. This resistance limits the efficacy of conventional therapies and underscores the need for novel treatment strategies that target EMT-associated pathways.

EMT also plays a significant role in immune evasion by altering the expression of immune checkpoint molecules. Mesenchymal-like MPM cells exhibit increased expression of PD-L1, which inhibits T-cell-mediated immune responses and allows tumors to evade immune surveillance [18]. This has important implications for immunotherapy, as EMT-driven tumors may exhibit variable responses to immune checkpoint inhibitors. Understanding the relationship between EMT and immune evasion may help identify biomarkers that predict immunotherapy response and guide the development of combination therapies [18].

Another major challenge posed by EMT in MPM is its contribution to tumor recurrence. Residual mesenchymal-like tumor cells following initial therapy often exhibit stem-like properties, allowing them to survive and repopulate the tumor. This underscores the need for novel therapeutic strategies that specifically target EMT-driven pathways to overcome treatment resistance and improve long-term patient outcomes.

Emerging evidence suggests that EMT-associated markers could predict responsiveness to therapy [20]. For instance, high AXL or PD-L1 expression may indicate sensitivity to AXL inhibitors or immune checkpoint blockade, respectively [20]. Additionally, reduced levels of miR-200c, a known suppressor of EMT, have been linked to resistance to chemotherapy [20]. These biomarkers warrant further investigation in stratifying patients for tailored treatment strategies.

## 4. EMT-Related Diagnostic and Prognostic Biomarkers in MPM

EMT-related biomarkers have become essential tools for diagnosing and prognosticating MPM. These biomarkers help distinguish between epithelial and mesenchymal phenotypes, which can have implications for patient prognosis and treatment selection. Several biomarkers have been identified, including E-cadherin, vimentin, periostin, and AXL, which can be detected through immunohistochemistry, serum assays, or molecular techniques [1,2].

A loss of E-cadherin expression is a hallmark of EMT and is frequently observed in mesenchymal-like MPM tumors [21,22,23]. E-cadherin loss is associated with increased tumor invasiveness and resistance to apoptosis, making it a potential prognostic biomarker for aggressive disease [21,22,23]. Conversely, vimentin, a mesenchymal marker, is overexpressed in sarcomatoid MPM and correlates with increased metastatic potential [8]. The upregulation of vimentin serves as an indicator of an EMT-driven phenotype and may guide treatment strategies that target mesenchymal-like tumor cells.

Periostin, a secreted extracellular matrix protein, has been implicated in tumor aggressiveness and has been proposed as a potential serum biomarker for MPM [2,24]. Studies suggest that periostin levels are elevated in patients with mesothelioma compared to healthy individuals, making it a promising candidate for early detection. Similarly, AXL, a receptor tyrosine kinase involved in EMT, has been identified as a marker of poor prognosis. AXL expression correlates with enhanced tumor cell migration and invasion, and its inhibition is being explored as a potential therapeutic approach in EMT-driven MPM.

MicroRNAs (miRNAs) have also emerged as key regulators of EMT in MPM, with certain miRNAs, such as the miR-200 family and miR-205, playing roles in maintaining epithelial integrity [25,26]. The downregulation of these miRNAs is commonly observed in sarcomatoid and biphasic MPM, further emphasizing their role in the EMT process [25,26]. The diagnostic and prognostic significance of miRNAs is being actively explored, with ongoing research investigating their potential as circulating biomarkers for non-invasive disease monitoring.

## 5. Emerging Therapeutic Strategies Targeting EMT in MPM

EMT has become an attractive target for novel therapeutic interventions in MPM. Table 3 demonstrates the primary clinical implications of EMT in MPM. Several experimental approaches aim to reverse EMT or exploit the vulnerabilities of EMT-driven tumors [27]. Among these strategies, TGF-β inhibitors have gained significant attention due to their ability to suppress EMT signaling. Agents such as fresolimumab and OT-101 (trabedersen) are currently under investigation to block TGF-β-driven EMT and improve patient outcomes [27,28].

FAK inhibitors, such as defactinib (VS-6063), have been explored as potential EMT-targeting agents due to their role in regulating mesenchymal cell survival [29]. However, clinical trials evaluating FAK inhibitors in MPM have yielded mixed results, with some studies failing to demonstrate significant clinical benefit. Nevertheless, combination therapies incorporating FAK inhibition with immunotherapy or chemotherapy are being investigated to enhance treatment efficacy [29,30].

AXL inhibitors represent another promising avenue for EMT-targeted therapy in MPM [30]. Given the strong correlation between AXL expression and EMT activation, inhibitors such as bemcentinib and cabozantinib are being tested in clinical trials. These agents aim to block AXL signaling and restore tumor sensitivity to conventional treatments, thereby improving patient outcomes [31,32].

Additionally, epigenetic therapies and microRNA-based treatments are being developed to reverse EMT and reprogram tumor cells toward a less aggressive phenotype [33]. Stratifying patients based on molecular EMT status may identify subgroups more likely to respond to combination therapies [34,35]. For example, patients with mesenchymal features may benefit from dual inhibition strategies targeting EMT pathways alongside immunotherapy. Integrating such stratification into clinical trial design could improve treatment personalization and efficacy [34,35]. Histone deacetylase (HDAC) inhibitors, for example, may help modulate EMT-associated gene expression, while miR-200c and miR-205 mimics are being explored as potential agents to restore epithelial characteristics in mesenchymal-like tumor cells [33,36].

## 6. Recent Clinical Trials and the MARS 2 Study

Recent clinical trials have significantly shaped our understanding of mesothelioma treatment and the role of EMT in determining therapeutic response. One of the most impactful studies in this domain is the CheckMate 743 trial [37], which demonstrated that the combination of immune checkpoint inhibitors, nivolumab and ipilimumab, offers superior survival benefits compared to chemotherapy in non-epithelioid MPM. This finding suggests that EMT-driven tumors, which often exhibit mesenchymal features and immune evasion mechanisms, may benefit more from immunotherapy-based regimens than traditional chemotherapeutic approaches. While CheckMate 743 demonstrated superior outcomes in non-epithelioid MPM, it remains unclear whether the combination of nivolumab and ipilimumab would provide similar benefit in patients with epithelioid subtypes [38,39]. Given the heterogeneity of MPM and overlapping EMT features even within epithelioid tumors, extending these trials to broader histological contexts is warranted [38,39]. Other trials, such as the DREAM3R study [40], continue to investigate combinations of checkpoint inhibitors with chemotherapy to improve outcomes for patients with EMT-high tumors.

Beyond immunotherapy, EMT-targeted therapies have emerged as a promising area of research. Several clinical trials are evaluating inhibitors of EMT-related pathways, such as TGF-β, Wnt/β-catenin, and AXL [41]. Preclinical studies suggest that blocking these pathways may reverse EMT and restore tumor sensitivity to chemotherapy and immunotherapy. For instance, galunisertib, a TGF-β receptor kinase inhibitor, has shown potential in reversing EMT-driven tumor progression, and trials investigating its combination with immune checkpoint blockade are underway [42]. Similarly, AXL inhibitors, such as bemcentinib, are being explored for their role in targeting mesenchymal-like tumor cells that are resistant to conventional therapy [43].

The role of surgery in MPM treatment has also been debated, particularly following the results of the MARS 2 trial [44]. This study investigated whether extended pleurectomy/decortication (eP/D) surgery, when combined with chemotherapy, offers survival benefits compared to chemotherapy alone. The trial’s findings suggested that surgery did not provide a significant improvement in overall survival, leading to the reconsideration of surgical intervention in MPM management. While surgical debulking may still be beneficial for symptom control in select patients, the results emphasize the need for systemic therapies that effectively target the underlying biology of MPM, including EMT-associated pathways [45].

Moving forward, clinical trials must incorporate molecular and histological stratification to better identify patients who may benefit from specific treatment regimens. The integration of EMT biomarkers into clinical trial design could help predict response to immunotherapy, chemotherapy, and targeted therapies. Personalized treatment approaches, based on EMT status, may become the standard of care in the future, allowing for more precise therapeutic strategies and improved patient outcomes.

## 7. Conclusions

Epithelial–mesenchymal transition is a key driver of malignant pleural mesothelioma progression, influencing tumor invasiveness, metastasis, immune evasion, and therapeutic resistance. The intricate interplay between transcription factors, signaling pathways, and the tumor microenvironment contributes to the aggressive nature of MPM, making EMT a critical focus for research and therapeutic intervention. EMT-related biomarkers, including E-cadherin, vimentin, periostin, AXL, and miR-200, provide valuable diagnostic and prognostic insights, enabling a more personalized approach to treatment selection. Non-invasive detection methods, such as liquid biopsies, may further enhance early diagnosis and disease monitoring. As EMT confers resistance to standard treatments, targeting EMT-related pathways, including TGF-β, Wnt/β-catenin, and PI3K/Akt, holds promise for overcoming therapy resistance and improving patient outcomes. Small-molecule inhibitors, epigenetic modulators, and microRNA-based therapies are being investigated as potential EMT-targeting interventions, with ongoing clinical trials assessing their feasibility and efficacy in MPM.

Recent advances in immunotherapy, particularly through immune checkpoint inhibitors such as nivolumab and ipilimumab, have shown promise in non-epithelioid MPM, highlighting the influence of EMT status on treatment response. However, EMT-driven tumors often exhibit immunosuppressive features, necessitating combination strategies that integrate EMT-targeted approaches with immunotherapy to enhance treatment efficacy. Additionally, the MARS 2 trial has questioned the role of radical surgery in MPM, underscoring the need for individualized treatment decisions based on molecular tumor characteristics, including EMT status. Moving forward, continued research into EMT-driven therapeutic strategies, biomarker-guided treatment selection, and innovative immunotherapeutic combinations will be essential to advancing the management of MPM. Through an interdisciplinary approach that leverages molecular profiling, targeted therapy, and novel clinical trial designs, there is hope for improved survival outcomes and a more tailored, effective treatment paradigm for patients affected by this devastating disease.

Recent studies suggest that EMT is not a binary process but occurs along a dynamic spectrum within tumors. The concept of intra-tumoral EMT heterogeneity—where different regions of the same tumor exhibit varying degrees of epithelial or mesenchymal traits—adds complexity to MPM biology [46,47]. This heterogeneity may influence treatment resistance and recurrence, highlighting a need for spatial transcriptomic and single-cell studies to unravel EMT gradients within tumors.

## Figures and Tables

**Table 1:** Key EMT-related pathways in MPM.

Signaling Pathway	Key Components	Role in EMT
TGF-β	TGF-β1, SMAD2/3, PI3K/Akt	Promotes mesenchymal phenotype
Wnt/β-Catenin	β-Catenin, GSK-3β, TCF/LEF	Induces transcription of EMT genes
PI3K/Akt	PTEN, mTOR, TWIST1	Increases cell survival and migration

Abbreviations: EMT: epithelial-to-mesenchymal transition; MPM: malignant pleural mesothelioma.

**Table 2:** Representative EMT biomarkers in solid tumors, including MPM.

Biomarker	Type	Clinical Relevance
E-cadherin	Protein	Low levels indicate EMT activation
Vimentin	Protein	High expression in mesenchymal MPM
Periostin	Secreted protein	Associated with poor prognosis
AXL	Receptor tyrosine kinase	Correlates with aggressive tumor phenotype
miR-200 family	microRNA	Low expression promotes EMT

Abbreviations: EMT: epithelial-to-mesenchymal transition; MPM: malignant pleural mesothelioma; AXL: AXL receptor tyrosine kinase.

**Table 3:** Clinical implications of EMT in MPM.

Feature	Epithelial MPM	Mesenchymal (EMT) MPM
Histology	Epithelioid	Sarcomatoid
Invasiveness	Low	High
Response to Chemotherapy	Favorable	Poor
PD-L1 Expression	Low	High
Prognosis	Better	Worse

Abbreviations: EMT: epithelial-to-mesenchymal transition; MPM: malignant pleural mesothelioma; PD-L1: Programmed Death-Ligand 1.

## Data Availability

Data can be made available by the authors upon reasonable request.

## Abbreviations

The following abbreviations are used in this manuscript:

MPM	Malignant pleural mesothelioma
EMT	Epithelial–mesenchymal transition
